# Accuracy and Precision of Equine Gait Event Detection during Walking with Limb and Trunk Mounted Inertial Sensors

**DOI:** 10.3390/s120608145

**Published:** 2012-06-12

**Authors:** Emil Olsen, Pia Haubro Andersen, Thilo Pfau

**Affiliations:** 1 Department of Large Animal Sciences, Faculty of Health and Medical Science, University of Copenhagen, Hojbakkegaard Alle 5, 2630 Taastrup, Denmark; E-Mail: pia@life.ku.dk; 2 Department of Veterinary Clinical Sciences, The Royal Veterinary College, North Mymms, Hawkshead Road, North Mymms, Herts AL9 7TA, UK; E-Mail: tpfau@rvc.ac.uk

**Keywords:** gait events, locomotion, horse, Inertial Measurement Units, Inertial Magnetic Measurement Unit, accuracy, precision, method comparison, stride segmentation

## Abstract

The increased variations of temporal gait events when pathology is present are good candidate features for objective diagnostic tests. We hypothesised that the gait events hoof-on/off and stance can be detected accurately and precisely using features from trunk and distal limb-mounted Inertial Measurement Units (IMUs). Four IMUs were mounted on the distal limb and five IMUs were attached to the skin over the dorsal spinous processes at the withers, fourth lumbar vertebrae and sacrum as well as left and right tuber coxae. IMU data were synchronised to a force plate array and a motion capture system. Accuracy (bias) and precision (SD of bias) was calculated to compare force plate and IMU timings for gait events. Data were collected from seven horses. One hundred and twenty three (123) front limb steps were analysed; hoof-on was detected with a bias (SD) of −7 (23) ms, hoof-off with 0.7 (37) ms and front limb stance with −0.02 (37) ms. A total of 119 hind limb steps were analysed; hoof-on was found with a bias (SD) of −4 (25) ms, hoof-off with 6 (21) ms and hind limb stance with 0.2 (28) ms. IMUs mounted on the distal limbs and sacrum can detect gait events accurately and precisely.

## 1. Introduction

When a horse is diagnosed with cervical vertebral spinal cord compression it often leads to euthanasia, as affected horses are considered unsafe to handle and ride due to their impaired coordination. At least one in 100 European horses are ataxic due to compression of the cervical spinal cord [1]. Compression of the spinal cord can interrupt the central pattern generator and reflexes in the spinal cord responsible for maintaining a rhythmic gait and coordination based on feedback from sensory and proprioceptive pathways [2]. The change in coordination is quantifiable as an increased variation in spatial and temporal gait characteristics [2,3]. Clinically, the moderately ataxic horse can be recognised during walk by applying various postural proprioceptive and coordination challenge tests assessing gait variability and consistency [4]. However diagnosing subtle ataxia is likely as difficult as diagnosing subtle lameness, where even experienced clinicians frequently disagree when deciding on the most affected limb [5] and where experience is crucial for intra-assessor consistency [6].

While ambulatory kinematic systems have been developed for use in lameness work-up in horses [7–11], no ambulatory systems are available for quantification of ataxia. In addition, only a limited number of studies have investigated spatiotemporal gait characteristics in the ataxic horse and are based on data obtained on the treadmill using kinematic cameras [12,13]. While the treadmill is a proven tool to obtain large numbers of strides, it affects kinematics [14,15]. Further, the treadmill decreases the variation in spatiotemporal gait characteristics [16], which could mask subtle changes in consistency of the gait events hoof-on/off in the ataxic horse. Using a single force plate an increased variability and magnitude of the lateral ground reaction force has been found in the ataxic horse [17]. Also fuzzy clustering of vertical position estimates of a reflective marker on the fetlock joint and mid lumbar dorsal spinous processes have been shown to correctly classify a horse as having spinal ataxia [13]. However, none of these methods are feasible for use in ambulatory practice, due to the expensive instrumentation and the need for a dedicated indoor gait lab.

Affordable inertial measurements units (IMUs) capable of collecting large amounts of data are now available. These IMUs provide an opportunity to collect stride series with the animal moving with fewer constraints than in a gait lab or on a treadmill. This provides an option for objective analysis of neurologic disorders with subtle changes in spatiotemporal gait characteristics and applies the information to evidence based clinical decision-making. However none of the currently commercially available IMUs have been validated for this demanding application, which requires the instrumentation to be portable, synchronised and remain calibrated for the high accelerations observed during equine hoof-to-ground impact [18]. Currently available, synchronizable, 6DoF IMUs are limited to recording accelerations of 18 g (g: acceleration due to gravity). Hoof accelerations frequently exceed these [18], hence, the most distal location for accurate movement quantification using IMUs is the fetlock.

This study aims to provide evidence supporting the use of distal limb mounted IMUs for the measurement of temporal gait events. The objective is to quantify the accuracy and precision of the gait events hoof-on/off based on IMUs mounted on the distal metacarpus (DMC) or distal metatarsus (DMT), withers, L4, sacrum and tuber coxae, using raw, rotated, rotated and filtered or rotated and integrated IMU data features. In particular, we hypothesise that data features (*i.e.*, local peaks, maxima or minima) matching the gait events hoof-on/off and stance can be derived with high accuracy and precision from DMC/DMT/withers/L4/sacrum/tuber coxae mounted IMU data streams for both front and hind limbs, compared to force plates as the reference (“gold”) standard.

## 2. Methods

### 2.1. Horses

Seven horses of different breeds were used in this study: four research Thoroughbreds, two client-owned Warmblood horses and one client-owned pony of unknown breed. There were five geldings and two mares with a mean age of 4.3 years (range 2 to 7 years), mean height of 1.56 m (range 1.35 to 1.69 m). Six horses had front limb shoes, one had hind limb shoes and one had no shoes. Four to six experts subjected the horses to a neurologic and lameness assessment at the Equine Referral Hospital at the Royal Veterinary College (RVC). Three of the horses were mildly lame and three were mildly to moderately ataxic. All procedures were carried out at the RVC Structure and Motion gait laboratory, approved by the ethics and welfare committee at the RVC and complied with the European Animal (Scientific Procedures) Act 1986.

### 2.2. Data Acquisition

A customised boot (SMBII, Professional's Choice Sports Medicine Products, El Cajon, CA, USA) was placed on each limb. The boots were modified with Velcro on the outside of the boots' tightening straps. An 18 g IMU (MTx, Xsens Technologies B.V, Enschede, The Netherlands) was fixed in a tight pocket with Velcro on both sides and strapped snuggly to the lateral side of the boot using Velcro. The IMU was located at the level of the distal end of the 4th metacarpal bone (MCIV). The right and left IMU were aligned to the same height from the ground using a laser distance measurement device (Disto D3, Leica Geosystems A/S, Herlev, Denmark).

**Figure 1.**
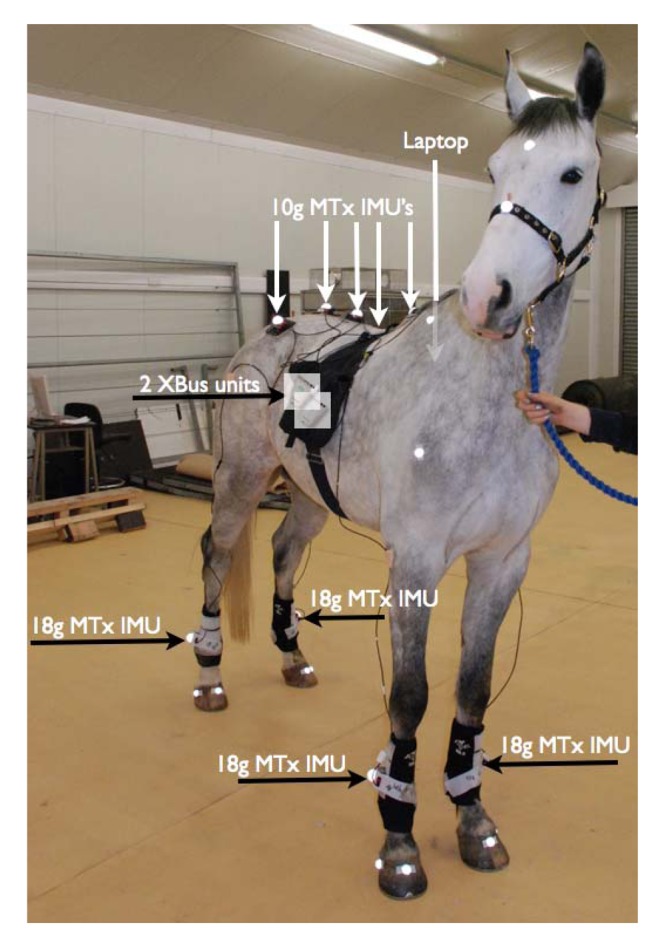
Distal limb mounted IMUs strapped onto a standard protective boot using Velcro. The IMUs are connected in series to two XBus Masters on the right side of the horse. The XBus Masters are plugged into a laptop mounted on the left side of the horse. Reflective markers are placed on the proximal dorsal and lateral hoof of each leg and on each of the IMUs.

Five 10 g IMUs were placed on the dorsal midline over: (1) the withers (2) the 4th lumbar dorsal spinous process (3) the most dorsal point on the spinous processes of the sacrum and (4 + 5) bilaterally over each tuber coxae. In one horse, four 10 g IMUs were used on the limbs. The dorsum sensors were fixed to the skin with custom made pockets and self-adhesive plaster (Animal Polster, Snøgg Industri AS, Kristiansand, Norway). A cable from each IMU was attached to a data streaming and controlling Xbus Master (Xsens) with a maximum of 5 IMUs per Xbus Master. The two Xbus Masters were connected with a RS232 cable to the USB port of a solid-state drive adapted 10.1” laptop (S10, Lenovo Technology, Hook, UK). The laptop was collecting data at 200 Hz using MT Manager software (Xsens) and remote-controlled via WIFI using dedicated software (TeamViewer GmbH, Goppingen, Germany). The setup is depicted in Figure 1; data collection was initiated and ended with 5 seconds of the horse standing still for each trial, to optimise performance of IMU orientation estimation.

An experienced handler walked the horses at their preferred walking speed along a 25 m runway with a centrally and seamlessly embedded 4.8 m × 0.9 m force plate array (8 × Kistler type 9287BA, Kistler Instrumente AG, Winterthur, Switzerland) where the force data were collected through a custom built 10-plate Interface Rack based on a Data Acquisition unit (NI-6225, National Instruments, Austin, TX, USA) filtered through a low-pass filter (−6 dB point of 100 Hz) and acquired in LabView (v. 8.6, National Instruments).

A 12-camera optical motion capture system (Qualisys Oqus 300 and 500 series, Qualisys AB, Gothenburg, Sweden) was synchronized to the force plates and used to collect 3D kinematic data. The force plates were sampling at 500 Hz and were down sampled using MATLAB (R2011a, The MathWorks Inc., Natick, MA, USA) to the 200 Hz-sampling rate of motion capture and IMUs.

For synchronisation with the force plates, all limb mounted IMU's had a 36 mm reflective marker placed over the centre of the sensor. A 26 mm reflective marker was attached to the proximal dorsal and lateral hoof wall on each leg. The left front leg IMU was tapped with a stick on which a 36 mm reflective marker was glued. The peak in the sensor's z-axis (approximately aligned with the horse's latero-medial axis) acceleration was used to synchronise the IMUs to the force plates with the start of the movement of the reflective marker.

### 2.3. Data Processing

IMU data were processed with automated custom written MATLAB scripts. A horse-based global right-handed Cartesian coordinate system was defined with the positive x-axis pointing in the walking direction of the horse, the positive y-axis pointing to the left side of the horse and the positive z-axis pointing upwards. IMU data consisted of three-dimensional (3D) acceleration, 3D angular velocity, 3D magnetic registration and orientation. Acceleration and angular velocity were rotated into the global horse coordinate system and integrated as described by Pfau *et al.* [19] with the modification of a 0.5 Hz cut-off for the high pass 4th order Butterworth filter applied before rotation into the global horse coordinate system (hereafter referred to as rotation). Data streams in the local sensor coordinate system included in the analyses were: 3D acceleration before and after filtering, magnetic sensing and orientation (roll, pitch and yaw). Data streams in the global horse coordinate system included in the analyses were: 3D acceleration; 3D angular velocity; 3D velocity and 3D displacement.

Force plate and kinematic data were analysed using a semi-automated custom written MATLAB script. Reflective hoof marker position was used to correctly classify which limb the force plate events related to. The beginning and end of stance was obtained applying a threshold of 10 N to the vertical force signal defining hoof-on/off.

### 2.4. Gait Event Detection

**Figure 2.**
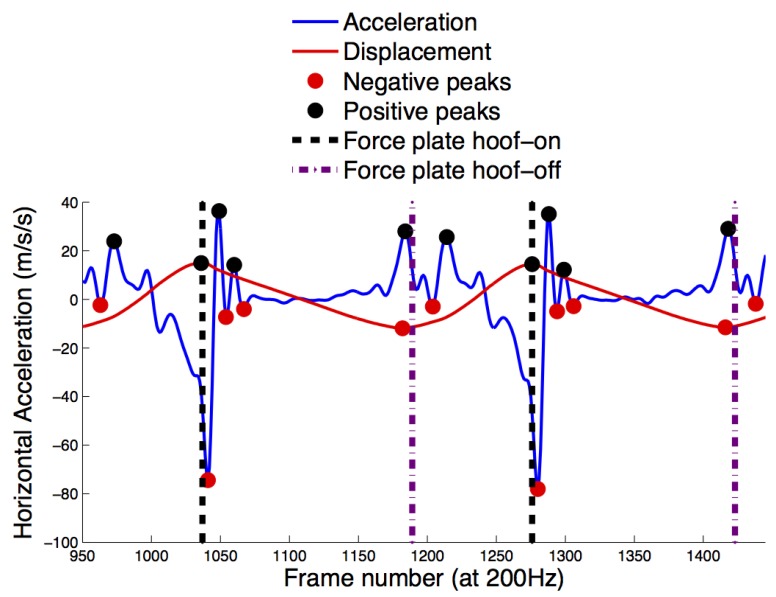
Example of gait event detection algorithm. This figure illustrates how the algorithms extract gait events from distal metatarsal IMU features. The red line is the cranio-caudal displacement of the IMU (displacement data multiplied by 30 and plotted on the acceleration axis) where the positive peaks (black dots) are guidelines for the approximate time of hoof-on. The negative peaks (red dots) are guidelines for the approximate time of hoof-off. The black interrupted vertical lines illustrate the timing for hoof-on based on force plates and the purple interrupted lines are the timing for hoof-off based on force plates. The negative peaks (red dots) on the blue cranio-caudal acceleration are matching hoof-on and are easily extracted as absolute local minima whereas the positive peaks (black dots) on the blue cranio-caudal acceleration are matching hoof-off, and can be extracted as the first local positive peak around the negative peak in displacement.

The first two horses were used as a template for the development of prototype gait event algorithms. Based on a frequency analysis, low pass Butterworth filters as well as wavelet symelets decomposition filters applied to each of the data streams were set as listed in Supplementary Table 1. Figure 2 is an example of how the local maxima and minima in the sinusoidal horizontal displacement of the DMC IMU were used as a guide for extraction of features in each of the signals.

### 2.5. Statistical Analysis

The statistical analysis was carried out using R version 2.13.1 [20] with the packages lattice [21] for graphics, pastecs [22] for descriptive statistics, psy [23] for Intra Class Correlation (ICC) and a function for agreement analysis with repeated measures [24] where gait events are assumed to be repeated dependent measures on the independent items horses. Agreement analysis corrected for repeated measurements on each horse was performed with accuracy defined as the mean difference (bias) between the methods with upper and lower limits of agreement as described by Bland and Altman [25]. Precision was defined as the global SD of the bias and percentage of error calculated as described by Metzelder *et al.* [26]. The ICC was calculated according to Schrout and Fleiss [27]. Stance was calculated combining the bias corrected hoof-on/off results. Algorithms detecting fewer than 95% of all steps in all horses were not further investigated.

## 3. Results and Discussion

Visual inspection of the data streams plotted with the force plate foot contact times (Figure 2) revealed that 34 data streams had consistent features that could be automated to extract front and hind limb hoof-on timings, 24 for front limb hoof-off and 30 for hind limb hoof-off (supplementary Table 1). For the front limbs, 123 stance phases with a mean stance time of 760 ms were included in the analysis. For the hind limbs, 119 stance phases were included with a mean stance time of 762 ms. Table 1 shows the agreement analysis, accuracy (bias), precision (SD), median and SE of the mean for the features extracted for the front limb and Table 2 the hind limb. The ICC for both front and hind limb hoof on/off were above 0.99 indicating excellent agreement while the ICC for stance ranged from 0.84 to 0.90 indicating good agreement between force plates and IMU data streams. The proportional error expressed as percentage of error for stance time duration was 10% for all front limb stance phase algorithms and 7% for the best hind limb stance detection algorithms ranging to 10% for the one with the poorest performance. The DMC/DMT mounted IMUs were more accurate at gait event detection than the IMUs at the withers, sacrum, lumbar and tuber coxae although the sacrum mounted IMU had good accuracy and precision for hind limb hoof-on as per Table 2. The MATLAB algorithms used to extract gait events from the IMUs are supplied online as supplementary material for this paper.

## 4. Conclusions and Outlook

### 4.1. Ambulatory Measurements *vs.* Gait Lab and Treadmill

Biomechanical analysis is increasingly applied as a paraclinical diagnostic tool in human [28] and veterinary medicine [9,10,17,29,30]. Objective recognition of normal repeatable movement patterns in horses requires at least 3–5 consecutive strides [31]. The stride length of a horse and e.g., the limited extent of the calibrated area of 3D motion capture systems and/or the restricted size of standard force platforms means that even the most advanced comparative gait laboratories only allow recording of three to four consecutive strides during over ground walk and trot. The use of an instrumented treadmill increases the number of consecutive strides [32,33]. However surface properties of the treadmill belt are different to “natural” equestrian surfaces and treadmills have been shown to alter gait parameters during walk resulting in a decreased step frequency, increased step length, increased stance time and less vertical movement of the hoofs [14,15]. In humans, treadmill walking and running result in a significantly longer stride time and increased stability of trunk acceleration characterised by Lyapunov exponents of state space representations [16,34]. In addition, at least two habituation sessions are required for trot and nine for walk to obtain reproducible kinematic variables for a horse on the treadmill [35], making the instrumented treadmill comparatively impractical as a paraclinical diagnostic tool. Therefore there is a need for accurate and precise kinematics with the capacity to record many strides for objective assessment of the ataxic horse.

### 4.2. Accurate and Precise Detection of Temporal Gait Events

To the authors' knowledge there are no reports concerning the use of gyroscopes, accelerometers or IMUs in other anatomical locations than the hoof for determining gait event timings in the horse. Algorithms using the sacrum, hip, thigh, shank and ankle have successfully been developed in humans [36] and hoof-on detected using IMUs on the sacrum for horses during trot [37]. Gyroscopic sensors were applied in a study of hoof break-over but not correlated to gold standard gait event timings, e.g., derived from force platforms [38]. A study using hoof-mounted accelerometers by Witte *et al.* [18] obtained a mean absolute error of 2.4 ms for front hoof-on during walk and 3.6 ms for front and hind limb hoof-off. The accuracy reported here, using vertical velocity for front hoof-on (bias: 2.7 ms), and horizontal acceleration for front hoof-off (bias: 0.7 ms) indicates a better accuracy for hoof-off and similar accuracy for hoof-on using distal limb-mounted IMUs compared to uni-axial accelerometers. It is likely that the precision of hoof-mounted accelerometers is better compared to the fetlock-mounted IMUs indicated by the low interquartile range reported by Witte *et al.* [18], however a direct comparison would require the SD of the difference between the methods. In addition Peham *et al.* [39] described a method (based on motion capture and the horizontal velocity of a dorsal hoof marker), which was found to have an average error (accuracy) for stance phase duration of 10.8 ms. The DMC/DMT mounted IMUs in this study have a better accuracy for stance phase detection (0.01–1.3 ms bias).

The vibration damping properties of the flexor tendons and the distal foot [40,41] result in a decrease of the impact-accelerations of the DMC/DMT relative to the hoof [41] and could decrease the accuracy and precision using this location for gait event detection. However there is good correlation between the metacarpophalangeal joint angle and the vertical ground reaction force [42] indicating that the resulting forces could be measured at the DMC/DMT level as shown by the high accuracy and precision of both the horizontal and vertical acceleration and velocity to detect hoof-on/off at the DMC/DMT level.

The good accuracy (3 ms), precision (36 ms) and limits of agreement (−11 to 17 ms) for vertical velocity of the sacrum to detect hoof-on was expected based on a sacrum mounted IMU in non-lame and lame horses during trot [37] indicating that we can obtain accurate and precise stride but not hoof-off, step or stance duration from sacrum withers, lumbar or tuber coxae mounted IMUs. A sacrum mounted IMU applied to humans [43,44] found a lower bias for stride step and stance duration (ranging from 2 μs to 2 ms) and similar limits of agreement (−15 ms to 25 ms) [43] compared to the present study.

### 4.3. Intra- and Inter-Horse Variation

The inter-individual variation is contributing to a higher SD but a low intra-individual variation, for which the repeated measures agreement analysis corrects [25]. The wider limits of agreement for stance phase can therefore be attributed to an increased intra-horse variation.

The 10 N cut-off for hoof-contact in the vertical ground reaction force is low for a horse, but similar to what has been found in humans [45]; we found that a higher cut-off on average moves the time for hoof-on one frame later (at 200 Hz) and the time for hoof-off one frame earlier increasing the bias. The 10 N cut-off is therefore closer to the kinematic gait events but could increase the SD due to the oscillations in the force trace after impact. Future validation studies could use a percentage of body weight or a percentage of peak vertical ground reaction force to remove the likely effect of different mass of the subjects. Further work should focus on accuracy and precision for 3D position estimate in walk and trot using limb mounted IMUs to add robust spatial parameters of the distal limbs to the set of parameters that can be quantified with IMUs during over ground locomotion in horses.

### 4.4. Conclusions

We demonstrate good accuracy and precision for detection of the gait events front and hind hoof-on/off using DMC/DMT and sacrum mounted IMUs in horses during walk. Stance phase duration is highly accurate but less precise. Based on these findings development of a portable system for spatiotemporal gait analysis in the horse seems promising for objective assessment of ataxia.

## Supplementary Material



## Figures and Tables

**Table 1.** Results from agreement analysis and descriptive statistics for front limb gait events. Displaying the three algorithms with the best (smallest) accuracy and precision, all based on features from DMC ^A^/DMT ^B^ mounted IMUs ^C^. All values are in ms. Values in bold have the best (smallest) accuracy and precision. The full table can be accessed online as supplementary material.

**Front Limb**		**Agreement**				**Descriptive Statistics**		

**Gait Event**	**Feature**	**LLoA** ^1^	**ULoA** ^2^	**Bias**	**ICC** ^3^	**Median Error**	**SE Mean**	**SD**
**Hoof-on**	XYZ Acceleration magnitude	−7.46	18.33	5.44	0.9993	28	2.90	32.30
	**Horizontal velocity**	**−16.52**	**1.77**	**−7.38**	**0.9992**	**−38**	**2.06**	**22.95**
	Vertical Velocity	−16.31	10.84	−2.73	0.9995	−20	2.98	33.20

**Hoof-off**	XYZ Acceleration magnitude	−16.46	14.41	−1.02	0.9994	5	3.53	39.20
	Horizontal acceleration ^4^	−14.36	16.78	1.21	0.9994	5	3.59	39.84
	**Horizontal acceleration** ^5^	**−13.86**	**15.26**	**0.70**	**0.9994**	**10**	**3.35**	**37.17**

**Stance**	Hoof on; Horizontal velocity Hoof off; Acceleration vector	−77.06	76.31	-0.38	0.8384	8	3.54	39.09
	**Hoof on; Horizontal velocityHoof off; Horizontal acceleration** ^5^	**−73.80**	**73.75**	**−0.02**	**0.8391**	**5**	**3.40**	**37.54**
	Hoof on; Horizontal velocityHoof off; Latero-medial pitch	−73.71	73.30	−0.21	0.8515	12	3.40	37.55

A DMC: Distal MetaCarpus

B Distal MetaTarsus

C IMUs: Inertial Measurement Units

1 Lower limits of agreement

2 Upper limits of agreement

3 Intra Class Correlation

4 Before rotation

5 Before rotation, wavelet decomposed.

**Table 2.** Results from agreement analysis and descriptive statistics for hind limb gait events. Displaying the three algorithms with the best (smallest) accuracy and precision, all based on features from DMC ^A^/DMT ^B^ mounted IMUs ^C^. All values are in ms. Values in bold have the best (smallest) accuracy and precision. The full table can be accessed online as supplementary material.

**Hind Limb**		**Agreement**				**Descriptive Statistics**		

**Gait Event**	**Feature**	**LLoA** ^1^	**ULoA** ^2^	**Bias**	**ICC** ^3^	**Median Error**	**SE Mean**	**SD**
**Hoof-on**	Horizontal acceleration ^4^	−11.33	9.88	−0.73	0.9998	−5	2.45	26.60
	**Vertical acceleration** ^5^	**−13.50**	**5.96**	**−3.77**	**0.9997**	**−20**	**2.28**	**24.79**
	Vertical acceleration ^6^	−16.52	4.03	−6.25	0.9994	−30	2.38	25.89

**Hoof-off**	XYZ velocity magnitude	−3.69	15.98	6.15	0.9995	35	2.32	25.03
	Horizontal acceleration ^6^	−5.91	11.48	2.78	0.9998	20	2.05	22.04
	**Horizontal displacement**	**−2.08**	**14.42**	**6.17**	**0.9995**	**35**	**1.95**	**21.00**

**Stance**	Hoof-on: Vertical acceleration ^4^ Hoof-off: Horizontal acceleration ^6^	−54.39	54.66	0.13	0.9078	2	2.60	27.91
	**Hoof-on: Vertical acceleration** ^4^ **Hoof-off: Horizontal displacement**	**−54.13**	**54.52**	**0.20**	**0.9021**	**0**	**2.58**	**27.70**
	Hoof-on: Horizontal acceleration ^4^ Hoof-off: Horizontal displacement	−56.56	56.90	0.17	0.8971	−2	2.67	28.67

A DMC: Distal MetaCarpus

B Distal MetaTarsus

C IMUs: Inertial Measurement Units

1 Lower limits of agreement

2 Upper limits of agreement

3 Intra Class Correlation

4 Before rotation

5 Before rotation, wavelet decomposed

6 After rotation, before filtering.
