# Supplementary material for: Accuracy and Precision of Equine Gait Event Detection during Walking with Limb and Trunk Mounted Inertial Sensors
Source: Sensors (Basel). 2012 Jun 12;12(6):8145–56. doi: 10.3390/s120608145 (PMC3436021; doi:10.3390/s120608145)
Supplement: Supplementary file 1 [file sensors-12-08145-s001.pdf]

OPEN ACCESS

sensors

ISSN 1424-8220

www.mdpi.com/journal/sensors

Supplementary Information

Accuracy and Precision of Equine Gait Event Detection during Walking with Limb and Trunk Mounted Inertial Sensors. *Sensors* 2012, *12*, 8145-8156

Emil Olsen [1,2,*], Pia Haubro Andersen [1] and Thilo Pfau [2]

[1] Department of Large Animal Sciences, Faculty of Health and Medical Science, University of Copenhagen, Hojbakkegaard Alle 5, 2630 Taastrup, Denmark; E-Mail: pia@life.ku.dk

[2] Department of Veterinary Clinical Sciences, The Royal Veterinary College, North Mymms, Hawkshead Road, North Mymms, Herts, AL9 7TA, UK; E-Mail: tpfau@rvc.ac.uk

* Author to whom correspondence should be addressed; E-Mail: eo@life.ku.dk; Tel.: +45-2092-1986; Fax: +45-3533-2944.

Table 1. Filtering of data streams and the feature used for stride detection. X is cranio-caudal, Y is latero-lateral, Z is dorso-ventral in a right-handed horse based Cartesian coordinate system.

Sensor location	Front limb	Hind limb	Hoof On	Hoof Off	Data stream	Original signal	Axes	Feature (per stride)	Filter Type [1]	Filter cutoff/ level *
DMC/DMT	√[2]	√	√	√	Displacement		X		-	-
	√	-[3]<br>-	√	-			X	Local peak	Butterworth<br>Wavelet	20 Hz<br>level 3
	√	-<br>-<br>√<br>-	√	-<br>-<br>√<br>-	Velocity	+rotated [4]<br>+filtered [5]	Z	Local peaks<br>Absolute min<br>Local min peak<br>Local max peak	Butterworth<br>Wavelet<br>Wavelet	30 Hz<br>level3
	√	√	√	√		Magnitude	XYZ	Local peaks	Wavelet	FL [6]:level4<br>HL [7]: level3

Table 1. *Cont.*

Sensor location	Front limb	Hind limb	Hoof On	Hoof Off	Data stream	Original signal	Axes	Feature (per stride)	Filter Type [1]	Filter cutoff/ level *
DMC/DMT		-		√				On: Absolute minima<br>Off: Absolut maxima	Butterworth	FL: 15 Hz<br>HL: 30 Hz
	√	√	√	-	Angular velocity		Y	HL: Local peaks<br>Zero crossing		
		√		√				On: Zero crossing<br>Off: absolute minima<br>HL: local peaks	Wavelet	level4
	√	√	-	√		-rotated [8]<br>-filtered [9]		Local peaks	Butterworth	FL: 30 Hz<br>HL: 20 Hz
	√	√	√	√					Wavelet	level 3
	√	√	-	√		+rotated<br>-filtered	X	Local peaks	Butterworth	FL:30Hz<br>HL: 20Hz
	√	√	√	√					Wavelet	level 3
		√		√		+rotated<br>+filtered		Local peaks	Butterworth	FL:30Hz<br>HL: 20Hz
	√		√							
		√		√					Wavelet	FL: level4<br>HL: level3
		√		√	Acceleration	-rotated<br>-filtered			Butterworth	20 Hz
	√		√							
		√		-				Local peaks	Wavelet	FL: level4<br>HL: level3
		√				+rotated<br>-filtered			Butterworth	FL: 6 Hz<br>HL: 60 Hz
	√		√	√			Z			
		√							Wavelet	level 3
		√				+rotated<br>+filtered		On: Maxima<br>Off: local peaks	Butterworth	30 Hz
	√		√	√						
		√						Local peaks	Wavelet	level 3
	√	√	√	√		Magnitude	XYZ	Local peaks	Wavelet	level 4
	√	√	√	√	Orientation		X	Absolute minima & maxima	Butterworth	FL: 30 Hz<br>HL:<br>12 Hz,<br>6th order

Table 1. *Cont.*

Sensor location	Front limb	Hind limb	HoofOn	Hoof Off	Data stream	Original signal	Axes	Feature (per stride)	Filter Type [1]	Filter cutoff/ level *
DMC/DMT	-	-	-	-				Local peaks	Wavelet	level4
	√	√	√	√			Y	Absolute minima and maxima	Butterworth	FL: 30 Hz HL: 8 Hz 6th order
	√	-	√	√				Local peaks	Wavelet	level 4
	√	-	√	√			Z	Absolute minima and maxima	Butterworth	6th order lowpass, 5 Hz
	√	√	√	-		Magnitude	XYZ	Local peaks	Wavelet	level 4
Withers	√	-	√	√			X	Local peaks	Butterworth	Bandpass 0.1–20 Hz
		-			Velocity				Wavelet	level 4
	√	-	√	-			Z	Local peaks	Butterworth	Bandpass 0.35–5 Hz
	√	-	√	-		-rotated -filtered	X	Local peaks	Butterworth	Bandpass 0.35–5 Hz
		-		√	Acceleration				Wavelet	level 4
	√	-	√	-		+rotated	X	Local peaks	Butterworth	Bandpass 0.35–5 Hz
Tuber Coxae	-	√	√	√		-rotated	X	Local peaks	Butterworth	10 Hz
	-	√	√	√		-filtered			Wavelet	level3
	-	√	√	√		+rotated		Local peaks	Butterworth	10 Hz
	-	√	√	√		-filtered			Wavelet	level4
	-	√	√	-	Acceleration	-rotated -filtered		Local peaks	Butterworth	Bandpass 0.35–10 Hz
	-	√	√	-		+rotated -filtered	Z	Local peaks	Butterworth	Bandpass 0.35–10 Hz
	-	√	√	√	Velocity		Z	Local peaks	Butterworth	20 Hz

Table 1. *Cont.*

Sensor location	Front limb	Hind limb	HoofOn	Hoof Off	Data stream	Original signal	Axes	Feature (per stride)	Filter Type [1]	Filter cutoff/ level *
Sacrum	-	√	√	√	Acceleration	-rotated -filtered	X	Local peaks	Butterworth	10 Hz
	-	√	√	√					Wavelet	level3
	-	√	√	√		+rotated -filtered	Z	Local peaks	Butterworth	10 Hz
	-	√	√	√					Wavelet	level3
	-	√	√	-		-rotated -filtered		Local peaks	Butterworth	Bandpass 0.35–10 Hz
	-	√	√	√	Velocity	Magnitude	Z	Local peaks	Wavelet	level4
	-	√	√	√			XYZ	Local peaks	Wavelet	level3

1: All Butterworth filters are 4th order and low-pass unless stated and all wavelet decomposition filters are symlets 4 with the level stated. 2: Features in the data stream resembling hoof-on/off was found. 3: Features resembling hoof-on/off was not found. 4: Rotated from local sensor into global horse based reference system. 5: Filtered before rotation. 6: Front Limb. 7: Hind Limb. 8: Not rotated from local sensor into global horse based reference system. 9: Not filtered before rotation.

Full Results Tables:

Table 2. Agreement and descriptive statistics for all front limb gait events derived from DMC/DMT mounted IMUs. All values are in ms. Values in bold have the lowest accuracy and precision.

Front Limb		Agreement				Descriptive Statistics		
Gait Event	Feature	LCL [1]	UCL [2]	Bias	ICC [3]	Median Error	SE Mean	SD
Hoof-on	Acceleration vector	−7.46	18.33	5.44	0.9993	28	2.90	32.30
	**Horizontal velocity**	**−16.52**	**1.77**	**−7.38**	**0.9992**	**−38**	**2.06**	**22.95**
	Vertical Velocity	−16.31	10.84	−2.73	0.9995	−20	2.98	33.20
Hoof-off	Acceleration vector	−16.46	14.41	−1.02	0.9994	5	3.53	39.20
	Horizontal acceleration[4]	−14.36	16.78	1.21	0.9994	5	3.59	39.84
	**Horizontal acceleration[5]**	**−13.86**	**15.26**	**0.70**	**0.9994**	**10**	**3.35**	**37.17**
	Latero-medial pitch	−34.10	−3.45	−18.77	0.9959	−85	3.51	38.89
Stance	Hoof on; Horizontal velocity Hoof off; Acceleration vector	−77.06	76.31	−0.38	0.8384	8	3.54	39.09
	**Hoof on; Horizontal velocity Hoof off; Horizontal acceleration[5]**	**−73.80**	**73.75**	**−0.02**	**0.8391**	**5**	**3.40**	**37.54**
	Hoof on; Horizontal velocity Hoof off; Latero-medial pitch	−73.71	73.30	−0.21	0.8515	12	3.40	37.55

1: Lower limits of agreement, 2: Upper limits of agreement, 3: Intra Class Correlation, 4: Before rotation. 5: Before rotation, wavelet decomposed.

Table 3. Agreement and descriptive statistics for all hind limb gait events derived from DMC/DMT and sacrum mounted IMUs. Where nothing else is stated the features are DMC/DMT derived. All values are in ms. Values in bold have the lowest accuracy and precision.

**Hind Limb**		**Agreement**				**Descriptive Statistics**		
**Gait Event**	**Feature**	**LCL** [1]	**UCL** [2]	**Bias**	**ICC** [3]	**Median Error**	**SE Mean**	**SD**
Hoof-on	Acceleration vector	−12.36	13.45	0.54	0.9996	5	2.99	32.53
	Velocity vector	−6.91	15.48	4.28	0.9996	25	2.62	28.31
	Horizontal acceleration [4]	−11.33	9.88	−0.73	0.9998	−5	2.45	26.60
	Horizontal acceleration [5]	−12.88	17.35	2.24	0.9995	10	3.55	38.58
	Horizontal acceleration [6]	−12.50	12.06	−0.22	0.9997	−5	2.81	30.48
	Horizontal acceleration [7]	−12.66	16.00	1.67	0.9995	8	3.34	36.31
	Horizontal acceleration [8]	−12.66	16.00	1.67	0.9995	8	3.34	36.31
	Horizontal displacement	−6.99	14.16	3.58	0.9997	20	2.45	26.62
	**Vertical acceleration [5]**	**−13.50**	**5.96**	**−3.77**	**0.9997**	**−20**	**2.28**	**24.79**
	Vertical acceleration [6]	−16.52	4.03	−6.25	0.9994	−30	2.38	25.89
	Vertical acceleration [9]	−15.90	13.14	−1.38	0.9995	0	3.38	36.68
	Vertical velocity, sacrum [7]	−11.33	17.11	2.89	0.9995	13	3.32	36.03
Hoof-off	Velocity vector	−3.69	15.98	6.15	0.9995	35	2.32	25.03
	Horizontal acceleration [4]	−15.26	15.75	0.24	0.9995	15	3.64	39.07
	Horizontal acceleration [5]	−11.19	13.42	1.11	0.9997	10	2.90	31.21
	Horizontal acceleration [6]	−5.91	11.48	2.78	0.9998	20	2.05	22.04
	Horizontal acceleration [7]	−12.37	18.98	3.30	0.9994	15	3.67	39.48
	Horizontal acceleration [8]	−12.37	18.98	3.30	0.9994	15	3.67	39.48
	**Horizontal displacement**	**−2.08**	**14.42**	**6.17**	**0.9995**	**35**	**1.95**	**21.00**
	Latero-medial angular velocity	−16.94	5.27	−5.84	0.9995	−25	2.54	27.39
	Latero-medial pitch	−9.33	17.50	4.09	0.9995	30	3.09	33.24
	Vertical acceleration [4]	−18.15	13.89	−2.13	0.9995	−10	3.69	39.74
Stance	Hoof-on: Acceleration vector<br>Hoof-off: Velocity vector	−73.00	72.95	−0.02	0.8483	2	3.44	36.87
	Hoof-on: Velocity vector<br>Hoof-off: Velocity vector	−65.23	67.02	0.90	0.8661	1	3.14	33.56
	Hoof-on: Horizontal acceleration [4]<br>Hoof-off: Velocity vector	−65.87	65.81	−0.03	0.8747	1	3.10	33.20
	Hoof-on: Horizontal acceleration [6]<br>Hoof-off: Velocity vector	−72.28	72.27	−0.01	0.8618	3	3.37	36.10

Table 3. *Cont.*

Hind Limb		Agreement				Descriptive Statistics		
**Gait Event**	**Feature**	**LCL [1]**	**UCL [2]**	**Bias**	**ICC [3]**	**Median Error**	**SE Mean**	**SD**
Stance	Hoof-on: Horizontal displacement<br>Hoof-off: Velocity vector	−63.74	63.86	0.06	0.8757	−3	3.01	32.32
	**Hoof-on:** Vertical acceleration [4]<br>Hoof-off: Velocity vector	−59.39	59.25	−0.07	0.8869	0	2.83	30.31
	**Hoof-on:** Vertical acceleration [6]<br>Hoof-off: Velocity vector	−61.83	61.64	−0.09	0.8815	−2	2.92	31.33
	**Hoof-on:** Vertical velocity sacrum<br>Hoof-off: Horizontal acceleration [6]	−77.74	77.00	−0.37	0.8376	4	3.66	39.27
	Hoof-on: Velocity vector<br>Hoof-off: Horizontal acceleration [5]	−72.36	74.93	1.29	0.8488	−4	3.51	37.44
	Hoof-on: Horizontal acceleration [4]<br>Hoof-off: Horizontal acceleration [5]	−75.51	75.89	0.19	0.8497	1	3.57	38.27
	Hoof-on: Horizontal displacement<br>Hoof-off: Horizontal acceleration [5]	−73.51	74.06	0.28	0.8503	−3	3.49	37.40
	Hoof-on: Vertical acceleration [4]<br>Hoof-off: Horizontal acceleration [5]	−69.30	69.60	0.15	0.8607	1	3.31	35.50
	Hoof-on: Vertical acceleration [5]<br>Hoof-off: Horizontal acceleration [5]	−75.69	75.94	0.12	0.8434	−2	3.57	38.27
	Hoof-on: Acceleration vector<br>Hoof-off: Horizontal acceleration [6]	−71.10	71.46	0.18	0.8620	−1	3.35	35.92
	Hoof-on: Velocity vector<br>Hoof-off: Horizontal acceleration [6]	−62.02	64.36	1.17	0.8832	2	3.00	32.01
	Hoof-on: Horizontal acceleration [4]<br>Hoof-off: Horizontal acceleration [6]	−62.68	63.02	0.17	0.8914	2	2.94	31.53
	Hoof-on: Horizontal acceleration [6]<br>Hoof-off: Horizontal acceleration [6]	−69.49	69.88	0.19	0.8775	10	3.23	34.63
	Hoof-on: Horizontal displacement<br>Hoof-off: Horizontal acceleration [6]	−60.54	61.06	0.26	0.8927	−1	2.86	30.66
	Hoof-on: Vertical acceleration [4]<br>Hoof-off: Horizontal acceleration [6]	−54.39	54.66	0.13	0.9078	2	2.60	27.91
	Hoof-on: Vertical acceleration [6]<br>Hoof-off: Horizontal acceleration [6]	−59.87	60.09	0.11	0.8950	0	2.81	30.18
	Hoof-on: Vertical velocity sacrum<br>Hoof-off: Horizontal acceleration [6]	−74.28	73.95	−0.17	0.8565	6	3.50	37.56

Table 3. *Cont.*

Hind Limb		Agreement				Descriptive Statistics		
Gait Event	Feature	LCL [1]	UCL [2]	Bias	ICC [3]	Median Error	SE Mean	SD
Stance	Hoof-on: Acceleration vector<br>Hoof-off: Horizontal displacement	−69.73	70.21	0.24	0.8571	2	3.29	35.29
	Hoof-on: Velocity vector<br>Hoof-off: Horizontal displacement	−60.04	62.63	1.29	0.8813	1	2.91	31.02
	Hoof-on: Horizontal acceleration [4]<br>Hoof-off: Horizontal displacement	−60.06	60.53	0.23	0.8916	0	2.83	30.33
	Hoof-on: Horizontal acceleration [6]<br>Hoof-off: Horizontal displacement	−66.30	66.81	0.25	0.8791	3	3.09	33.15
	Hoof-on: Horizontal displacement<br>Hoof-off: Horizontal displacement	−58.26	58.91	0.32	0.8921	−3	2.76	29.56
	**Hoof-on: Vertical acceleration [4]<br>Hoof-off: Horizontal displacement**	**−54.13**	**54.52**	**0.20**	**0.9021**	**0**	**2.58**	**27.70**
	Hoof-on: Horizontal acceleration [4]<br>Hoof-off: Horizontal displacement	−56.56	56.90	0.17	0.8971	−2	2.67	28.67
	Hoof-on: Vertical acceleration [4]<br>Hoof-off: Horizontal displacement	−78.41	78.87	0.23	0.8211	−3	3.71	39.75
	Hoof-on: Vertical velocity sacrum [4]<br>Hoof-off: Horizontal displacement	−72.64	72.42	−0.11	0.8522	4	3.43	36.83
	Hoof-on: Velocity vector<br>Hoof-off: Latero-medial angular velocity	−73.16	75.66	1.25	0.8606	1	3.48	37.13
	Hoof-on: Horizontal acceleration [4]<br>Hoof-off: Latero-medial angular velocity	−76.65	76.68	0.02	0.8619	−4	3.52	37.79
	Hoof-on: Horizontal displacement<br>Hoof-off: Latero-medial angular velocity	−72.94	73.14	0.10	0.8673	−3	3.38	36.22
	Hoof-on: Vertical acceleration [4]<br>Hoof-off: Latero-medial angular velocity	−64.22	64.18	−0.02	0.8898	0	3.01	32.28
	Hoof-on: Vertical acceleration [6]<br>Hoof-off: Latero-medial angular velocity	−72.98	72.88	−0.05	0.8675	3	3.36	36.07
	Hoof-on: Vertical acceleration [4]<br>Hoof-off: Latero-medial pitch	−76.20	76.41	0.10	0.8460	6	3.55	38.06
	Hoof-on: Vertical acceleration [6]<br>Hoof-off: Latero-medial pitch	−79.29	79.45	0.08	0.8385	8	3.67	39.39

1: Lower limits of agreement, 2: Upper limits of agreement, 3: Intra Class Correlation, 4: Before rotation. 5: Before rotation, wavelet decomposed. 6: After rotation, before filtering. 7: After rotation and filtering, wavelet decomposed. 8: After rotation, before filtering, wavelet decomposed. 9: After rotation and filtering.

Table 4. Results from agreement analysis and descriptive statistics for each horse for front limb gait events. Displaying the three algorithms with the lowest accuracy and precision, all based on features from DMC [A]/DMT [B] mounted IMUs [C]. All values are in ms. Values in bold have the lowest accuracy.

Gait Event	Feature	Accuracy (Bias) by horse ID						
**Front Limb**		**A**	**B**	**C**	**D**	**E**	**F**	**G**
	XYZ Acceleration magnitude	−0.8	10.4	13.1	48.6	31.4	41.0	31.9
**Hoof-on**	**Horizontal velocity**	**−20.8**	**−40.2**	**−23.1**	**−27.4**	**−47.4**	**−28.0**	**−48.6**
	Vertical Velocity	25.8	−18.4	30.9	−8.2	−36.2	−34.0	−25.6
	XYZ Acceleration magnitude	19.2	−14.6	−11.6	13.8	−11.4	−4.0	−9.7
**Hoof-off**	Horizontal acceleration [1]	7.5	3.6	−0.63	9.0	7.8	30.0	1.7
	**Horizontal acceleration [2]**	**13.3**	**−3.8**	**−1.3**	**15.4**	**−2.1**	**1.0**	**8.3**
**Hind limb**								
	Horizontal acceleration [3]	0.4	0.2	−7.7	−1.3	−21.0	−22.0	19.7
**Hoof-on**	**Vertical acceleration [4]**	**−14.1**	**−22.8**	**−27.7**	**−12.1**	**−19.8**	**−35.0**	**−14.3**
	Vertical acceleration [5]	−24.6	−40.0	−15.9	−22.7	−45.0	−41.0	−22.7
	XYZ velocity magnitude	27.1	97.5	49.5	−54.6	84.2	13.0	18.0
**Hoof-off**	Horizontal acceleration [5]	37.1	33.5	46.8	−59.7	6.3	9.0	9.2
	**Horizontal displacement**	**7.9**	**4.8**	**−13.2**	**19.0**	**4.3**	**−54.0**	**−15.3**

A: DMC: Distal MetaCarpus; B: Distal MetaTarsus; C: IMUs: Inertial Measurement Units; 1: Before rotation; 2: Before rotation, wavelet decomposed. 3: Before rotation; 4: Before rotation, wavelet decomposed; 5: After rotation, before filtering.

© 2012 by the authors; licensee MDPI, Basel, Switzerland. This article is an open access article distributed under the terms and conditions of the Creative Commons Attribution license (http://creativecommons.org/licenses/by/3.0/).
